# Identification of Colorectal Cancer Related Genes with mRMR and Shortest Path in Protein-Protein Interaction Network

**DOI:** 10.1371/journal.pone.0033393

**Published:** 2012-04-04

**Authors:** Bi-Qing Li, Tao Huang, Lei Liu, Yu-Dong Cai, Kuo-Chen Chou

**Affiliations:** 1 Key Laboratory of Systems Biology, Shanghai Institutes for Biological Sciences, Chinese Academy of Sciences, Shanghai, People's Republic of China; 2 Shanghai Center for Bioinformation Technology, Shanghai, People's Republic of China; 3 Institute of Systems Biology, Shanghai University, Shanghai, People's Republic of China; 4 Centre for Computational Systems Biology, Fudan University, Shanghai, People's Republic of China; 5 Gordon Life Science Institute, San Diego, California, United States of America; Instituto Butantan, Brazil

## Abstract

One of the most important and challenging problems in biomedicine and genomics is how to identify the disease genes. In this study, we developed a computational method to identify colorectal cancer-related genes based on (i) the gene expression profiles, and (ii) the shortest path analysis of functional protein association networks. The former has been used to select differentially expressed genes as disease genes for quite a long time, while the latter has been widely used to study the mechanism of diseases. With the existing protein-protein interaction data from STRING (Search Tool for the Retrieval of Interacting Genes), a weighted functional protein association network was constructed. By means of the mRMR (Maximum Relevance Minimum Redundancy) approach, six genes were identified that can distinguish the colorectal tumors and normal adjacent colonic tissues from their gene expression profiles. Meanwhile, according to the shortest path approach, we further found an additional 35 genes, of which some have been reported to be relevant to colorectal cancer and some are very likely to be relevant to it. Interestingly, the genes we identified from both the gene expression profiles and the functional protein association network have more cancer genes than the genes identified from the gene expression profiles alone. Besides, these genes also had greater functional similarity with the reported colorectal cancer genes than the genes identified from the gene expression profiles alone. All these indicate that our method as presented in this paper is quite promising. The method may become a useful tool, or at least plays a complementary role to the existing method, for identifying colorectal cancer genes. It has not escaped our notice that the method can be applied to identify the genes of other diseases as well.

## Introduction

Colorectal cancer (CRC) is one of the most common malignancies in the western countries and a major cause of cancer-related death. Early detection of CRC could reduce the morbidity and improve the prognosis. Therefore, it is of great importance to identify cancer-related genes that could be used as biomarker for early diagnosis.

Recently, with the development of high-throughput biotechnologies, a large amount of biological data has been generated, such as yeast two-hybrid systems, protein complex and gene expression profiles, etc. These data are useful resources for deducing and understanding gene functions [1], [2], [3], [4], [5], [6], [7], [8]. So far the protein-protein interaction (PPI) data has been widely used for gene function prediction with the assumption that interacting proteins share the same or have similar functions and hence may be involved in the same pathway. This “guilty by association” rule was first proposed by Nabieva et al. [9] and can also be used to identify cancer related genes.

STRING is an online database resource which is an abbreviation for **S**earch **T**ool for the **R**etrieval of **I**nteracting **G**enes [10]. It provides both experimental as well as predicted interaction information with a confidence score. Algorithms based on PPI suggest that proteins with short distances to each other in the network are more likely to share the common biological functions [11], [12], [13], [14], and that interactive neighbors are more likely to have identical biological function than non-interactive ones [15], [16]. This is because the query protein and its interactive proteins may form a protein complex to perform a particular function or involved in a same pathway.

Although the successful application of the high-throughput data for gene function perdition and identification of novel genes associated with cancers, the errors in the high-throughput data have not been well solved yet. In this paper, we proposed a new method for identifying CRC related genes by integrating gene expression profile and a weighted functional protein association network constructed with PPI data from STRING. This method can make up the defect of only using high-throughput data. Meanwhile, the mRMR (**m**aximum **r**elevance **m**inimum **r**edundancy) algorithm [17] was utilized to identify six promising candidate genes distinguishing tumor and the normal colorectal samples. The Dijkstra's algorithm [18] was used to construct the shortest paths between each pair of the six genes. Moreover, additional 35 genes on these shortest paths were also identified and analyzed. For such 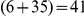 gene thus identified, it was observed that they contained more cancer genes than the genes identified from the gene expression profiles alone. Furthermore, the 41 genes also had greater functional similarity with the reported CRC genes than the genes identified from gene expression profiles alone. It is anticipated that some of the 41 genes thus identified might belong to novel CRC related genes.

## Materials and Methods

### Dataset

We used the gene expression data from the colorectal cancer study of Hinoue et al. [19]. The gene expression profiling of 26 colorectal tumors and matched histologically normal adjacent colonic tissue samples were retrieved from NCBI Gene Expression Omnibus (GEO) with the accession number of GSE25070. The gene expression profile was obtained using the Illumina Ref-8 whole-genome expression BeadChip with 24526 probes corresponding to 18491 genes. Signal intensity was log2 transformed and then normalized with RSN (**R**obust **S**pline **N**ormalization) method.

### Tissue sample representation

Based on the above, the representation of a tissue sample can be formulated as a 24526-D (dimensional vector), as given by
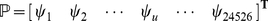
(1)where 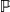 represents the tissue sample, 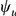 the value of it's 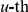 probe, and 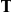 the transpose matrix (cf. Eq.6 of [20]).

### Cancer related gene list and two colorectal cancer related gene lists

We compiled three gene lists from public databases and published works to compare with the 41 candidate genes we identified. These three genes lists included one cancer related gene list and two colorectal cancer related gene lists.

742 cancer-related genes were derived from three sources. First, we obtained 457 cancer-related genes from the Cancer Gene Census of the Sanger Centre. Secondly, we retrieved cancer-related genes from the Atlas of Genetics and Cytogenetic in Oncology [21]. The third part was collected from the Human Protein Reference Database [22]. See Supporting Information S1.

The first colorectal cancer related gene list was retrieved from the study of Sabates-Bellver and coworkers [23]. They compared the transcriptomes of 32 adenomas with normal mucosa from the same individuals and identified 438 genes with markedly altered expression in colorectal adenomas compared with normal mucosa with Affymetrix U133 Plus 2.0 array. See Supporting Information S1.

The second colorectal cancer related gene list was retrieved form a recent work of Nagaraj et al. [24]. They proposed a Boolean based systems biology approach with guilt-by-association algorithm to identify novel cancer-associated genes. We compiled all the 134 novel CRC related genes identified in this study. See Supporting Information S1.

### PPI data from STRING

The initial weighted PPI network was retrieved from STRING (version 9.0) [10] (http://string.embl.de/), which is a large database of known and predicted protein interactions. Proteins in the interaction network were represented with nodes, while the interaction between any two proteins therein was represented with an edge. These interactions contain direct (physical) and indirect (functional) interactions, derived from numerous sources such as experimental repositories, computational prediction methods. In the network, each edge is marked with a score to quantify the interaction confidence, i.e., the likelihood that an interaction may occur.

### The mRMR (maximum relevance minimum redundancy) method

To find the genes that can distinguish colorectal tumors and normal adjacent tissues, we used the mRMR method, which was originally developed by Peng et al. [17] for analyzing the microarray data. The mRMR method could rank genes according to their relevance to the class of samples concerned, and meanwhile also could take the redundancy of genes into account. Those genes, which have the best trade-off between the maximum relevance to the sample class and the minimum redundancy, were considered as “good” biomarkers.

Both the relevance and redundancy were quantified by the following mutual information (MI):
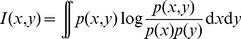
(2)where 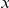 and 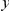 are vectors, 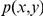 is their joint probabilistic density, and 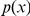 and 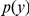 are the marginal probabilistic densities.

To quantify both the relevance and redundancy, let us define 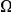 as the whole gene set, 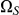 as the already-selected gene set containing 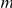 genes and 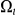 as the to-be-selected gene set containing 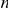 genes. The relevance 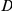 between the gene 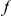 in 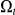 and the target 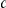 can be calculated by:
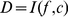
(3)The redundancy 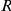 between the gene 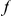 in 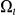 and all the genes in 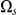 can be calculated by:
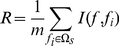
(4)In order to obtain the gene 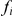 in 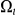 with the maximum relevance and minimum redundancy, let us combine Eq.3 and Eq.4, as can be formulated as follows:
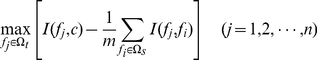
(5)Given a gene set with 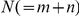 genes, the mRMR operation for the gene evaluation will continue 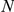 rounds. After these evaluations, the mRMR method will generate a gene set 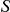 as formulated by
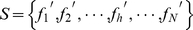
(6)where the index 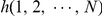 indicates which round the gene is selected. The smaller the index 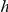 is, the earlier the gene satisfied Eq.5 and the better the gene is.

### Prediction engine

In this study, the Nearest Neighbor Algorithm (NNA) [25], [26], which has been widely used in bioinformatics and computational biology [3], [27], [28], [29], [30], [31], [32], [33], [34], was adopted to predict the class of colorectal tissue samples. The “nearness” was calculated according to the following equation
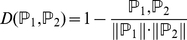
(7)where 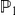 and 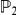 are two vectors representing two tissue samples, 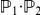 is their dot product, 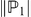 and 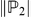 are their moduluses. The smaller the 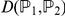, the more similar the two samples are [35]. For an intuitive illustration of how NNA works, see Fig.5 of [20].

### Performance validation

The following three cross-validation methods are often used in statistics for validating a statistical prediction method: independent dataset test, subsampling test, and jackknife test [36]. However, among the three validation methods, the jackknife test is the least arbitrary due to the following facts. (i) For the independent dataset test, although all the samples used to test the predictor are outside the training dataset used to train the prediction engine so as to exclude the “memory” effect or bias, the way of how to select the independent samples to test the predictor could be quite arbitrary unless the number of independent samples is sufficiently large. This kind of arbitrariness might lead to completely opposite conclusions. For instance, the conclusion that a predictor yielded a higher success rate than the other predictor for a given independent testing dataset might become just opposite when tested by another independent testing dataset [36]. (ii) For the subsampling test, the concrete procedure usually used in literatures is the 5-fold, 7-fold or 10-fold cross-validation. The problem with this kind of subsampling test is that the number of possible selections in dividing a benchmark dataset is extremely large even for a very simple and small dataset, as elucidated in [37] and demonstrated by Eqs.28–30 in [20]. Therefore, in any actual subsampling cross-validation tests, only a very tiny fraction of the possible selections are taken into account. Since different selections will always result in different outcomes even for a same benchmark dataset and a same predictor, the subsampling test cannot avoid the arbitrariness either. A test method unable to yield a unique outcome cannot be regarded as a good one. (iii) In the jackknife test, all the samples in the benchmark dataset will be singled out one-by-one and tested by the predictor trained by the remaining samples. During the process of jackknifing, both the training dataset and testing dataset are actually open, and each sample will be in turn moved between the two. The jackknife test can exclude the “memory” effect. Also, the arbitrariness problem as mentioned above for the independent dataset test and subsampling test can be avoided because the outcome obtained by the jackknife test is always unique for a given benchmark dataset. Accordingly, the jackknife test has been widely and increasingly used to inspect the quality of various predictors (see, e.g., [30], [31], [32], [38], [39], [40], [41], [42], [43], [44], [45], [46]). Accordingly, in this study the jackknife test was also used to examine the quality of the current prediction method.

The prediction accuracy was formulated by
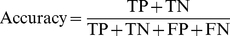
(8)where TP represents the true positive; TN, the true negative; FP, the false positive; and FN, the false negative.

### Incremental feature selection (IFS)

Based on the ranked genes according to their importance after mRMR evaluation, we used the Incremental Feature Selection (IFS) (see, e.g., [1], [47]) to determine the optimal number of genes as biomarkers. During the IFS procedure, genes in the ranked gene set are added one by one from higher to lower rank. A new gene set is composed when one gene is added. Thus 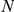 gene sets would be composed when given 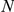 ranked genes. The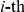 gene set is
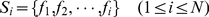
(9)For each of the N gene sets, an NNA predictor was constructed and examined using the jackknife test to the benchmark dataset. By doing so we obtained an IFS table with one column for the index *i* and another column for the prediction accuracy. Thus, we could obtain the optimal gene set (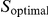), with which the predictor would yield the best prediction accuracy.

### Graph approach and shortest paths tracing

Graphs are a useful vehicle for studying complex biological systems because they can provide intuitive insights and the overall structure property, as demonstrated by various studies on a series of important biological topics (see, e.g., [48], [49], [50], [51], [52], [53], [54], [55], [56], [57], [58]). In this study, we first constructed a graph G(V, E) with the PPI data from STRING. In the graph, an edge was assigned for each pair of genes if they were in interaction with each other. The weight of edge E in graph G was derived from the confidence score according to the equation 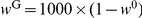, where 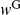 is the weight in graph G while 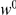 is the confidence score between two proteins concerned. Thus, we get a functional protein association network with edge weight. Dijkstra's algorithm [18] was used to find the shortest path from each of the six genes to all the other five genes in the graph. Then we picked out all the genes existing in the shortest paths and rank these genes according to their betweenness.

### KEGG enrichment analysis

Functional annotation tool of DAVID [59] was used for KEGG pathway enrichment analysis. The enrichment p-value was corrected to control family-wide false discovery rate under certain rate (e.g., ≤0.05) with Benjamin multiple testing correction method [60]. All the genes on the BeadChip were selected as background during the enrichment analysis.

## Results

### mRMR results

The expression profile was retrieved from GEO with the accession number of GSE25070, which contained 52 samples and 24,526 probes and was transformed to a CSV file with 52 rows and 24526 columns as the input of mRMR. Each probe represented a feature and the 26 tumor samples belonged to class 1 while the paired26 paired normal samples belonged to class 2. After running the mRMR software, we obtained two tables (see Supporting Information S2), of which one was called MaxRel table that ranked the probes according to their relevance to the class of samples, and the other called mRMR feature table that listed the probes with the maximum relevance and minimum redundancy to the class of samples.

### Six candidate genes identified by NNA and IFS

**Figure 1:**
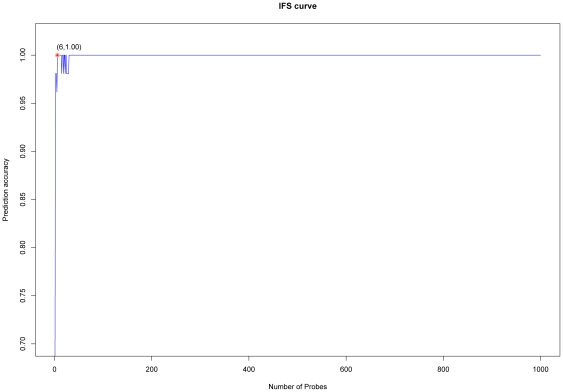
IFS curve for the colorectal tumors and matched normal adjacent tissue samples classification. In the IFS curve, the X-axis is for the number of probes used for classification, and the Y-axis for the prediction accuracies by the nearest neighbor algorithm (NNA) evaluated by the jackknife (Leave-One-Out) cross-validation test. The peak accuracy was 1 with six probes. The top 6 probes in the mRMR probe list formed the optimal discriminative probe set.

On the basis of the outputs of mRMR, we constructed 1000 feature subsets according to Eq.9. As described in the Materials and Methods section, we tested the predictor with one feature, two features, three features, etc., and the IFS result can be found in Supporting Information S3. Shown in **Fig. 1** is the IFS curve plotted based on the data of Supporting Information S3. In the IFS curve, the X-axis is the number of probes used for classification, and the Y-axis is the prediction accuracies of the nearest neighbor algorithm evaluated by the jackknife test. The maximum accuracy was 1 when 6 features were included. The optimal probe set included 6 probes corresponding to 6 different genes, which were GUCA2B, PI16, CDH3, SPIB, BEST2, and HMGCLL1 (**Table 1**).

**Table 1:** mRMR top six genes.

order	Probe name	Symbol	EntrezID	Protein ID
1	ILMN_1735578	GUCA2B	2981	ENSP00000361662
2	ILMN_1766264	PI16	221476	ENSP00000362778
3	ILMN_1704294	CDH3	1001	ENSP00000264012
4	ILMN_2143314	SPIB	6689	ENSP00000270632
5	ILMN_1755796	BEST2	54831	ENSP00000042931
6	ILMN_2339192	HMGCLL1	54511	ENSP00000381654

### Shortest paths genes

**Figure 2:**
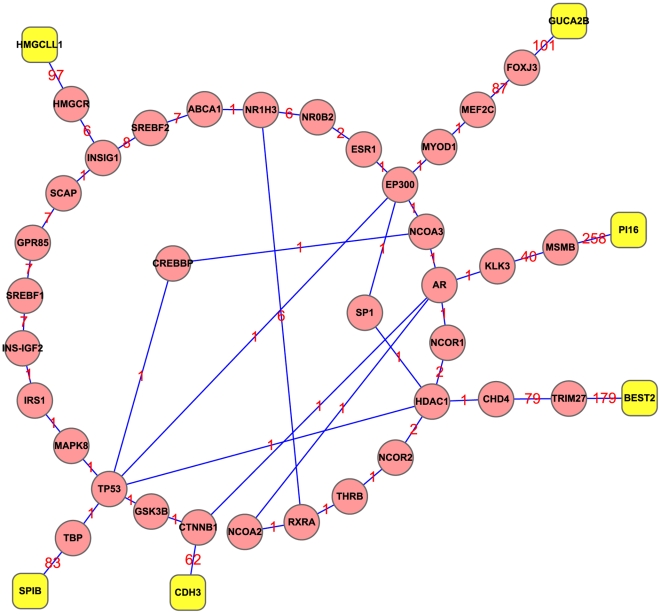
15 shortest paths between the six genes identified with mRMR method. The 15 shortest paths between the six candidate genes were identified with Dijkstra's algorithm based on the PPI data from STRING. Yellow roundrect represents the top six candidate genes identified by the mRMR method. Red round represents the 35 genes existing within the range of the shortest paths. Numbers on edges represent the edge weights to quantify the interaction confidence. The smaller the number is, the stronger the interaction between two nodes is. See the text in the Section of “Graph approach and shortest paths tracing” for the quantitative relation of the edge weight with the confidence score between two proteins concerned.

Meanwhile, we constructed an undirected graph with the PPI data from STRING. Then we picked out two genes from the six genes identified with the mRMR method as described above, and found out the shortest path between these two genes with the Dijkstra's algorithm. We obtained a total of 15 shortest paths with lowest cost (Supporting Information S4). Shown in **Fig. 2** are the 15 shortest paths between the six candidate genes, where the interaction confidence was labeled on the edge for each of the interaction gene pairs. There were a total of 35 genes on the shortest paths and we ranked these genes according to their betweenness (**Table 2**). Among these 35 genes, AR has the largest betweenness of 7, meaning that there are 7 shortest paths going through this gene. Accordingly, AR may play an important role in connecting the six candidate genes and hence may be related to CRC. Such a conclusion is fully consistent with the fact that AR protein was found in normal colorectal mucosa as well as in most CRC [61], [62], implying that the AR receptor is responsible for the mitogenic effects of the hormone as will be further discussed later.

**Table 2:** Shortest paths genes.

order	Protein id	symbol	betweenness	P-value
1	ENSP00000363822	AR	7	0*
2	ENSP00000269305	TP53	6	0.3442
3	ENSP00000230354	TBP	5	0.0066*
4	ENSP00000250003	MYOD1	5	0.0006*
5	ENSP00000263253	EP300	5	0.0598
6	ENSP00000287936	HMGCR	5	0*
7	ENSP00000314151	KLK3	5	0*
8	ENSP00000344456	CTNNB1	5	0.0984
9	ENSP00000344741	INSIG1	5	0*
10	ENSP00000349508	CHD4	5	0*
11	ENSP00000351363	MSMB	5	0*
12	ENSP00000354620	FOXJ3	5	0*
13	ENSP00000362649	HDAC1	5	0.0108*
14	ENSP00000396219	MEF2C	5	0*
15	ENSP00000417884	TRIM27	5	0*
16	ENSP00000342470	NR1H3	4	0.005*
17	ENSP00000354476	SREBF2	4	0.0038*
18	ENSP00000363868	ABCA1	4	0.0098*
19	ENSP00000361066	NCOA3	3	0.0038*
20	ENSP00000419692	RXRA	3	0.0098*
21	ENSP00000324806	GSK3B	2	0.1016
22	ENSP00000399968	NCOA2	2	0.0308*
23	ENSP00000206249	ESR1	1	0.1968
24	ENSP00000254227	NR0B2	1	0.0346*
25	ENSP00000262367	CREBBP	1	0.0754
26	ENSP00000265565	SCAP	1	0.0088*
27	ENSP00000268712	NCOR1	1	0.0176*
28	ENSP00000297146	GPR85	1	0.0104*
29	ENSP00000304895	IRS1	1	0.0976
30	ENSP00000329357	SP1	1	0.1242
31	ENSP00000348069	SREBF1	1	0.023*
32	ENSP00000348551	NCOR2	1	0.0162*
33	ENSP00000348827	THRB	1	0.0082*
34	ENSP00000348986	INS-IGF2	1	0.0898
35	ENSP00000353483	MAPK8	1	0.1194

* : P-value<0.05, significant.

To test whether our 35 shortest path genes were hubs in the background network or not, we ran a permutation to count the occurrence time of our 35 shortest path genes in the shortest paths between 6 random selected genes when they has greater betweenness than that in our study. We repeated this process 5000 times, and the p-value was calculated as the proportion of occurrence time of the 35 genes in 5000 permutation. For detail, please see **Table 2**. There were 10 shortest path genes whose p-values were not significant. TP53 was a star molecular involved in numerous biological processes and nearly related to all kinds of cancers [63]. Therefore, it is nothing surprising that TP53 appeared many times in shortest path between 6 randomly picked genes. For EP300, it has been reported that this gene can acetylate TP53 and associated with lots of tumors [64]. CTNNB1 and GSK3B belong to the Wnt signaling pathway, the role of which in caners has been well documented [65]. For the remaining insignificant 6 genes, their betweennesses in our study were all one (**Table 2**), and hence the number of occurrences for these genes in random shortest paths is prone to be greater than one. Most of these insignificant 6 shortest path genes fall behind in **Table 2** according to their betweennesses, suggesting that they might not be important. Besides these 10 genes, the remaining 25 shortest path genes in our study were identified to be significant.

### MaxRel table gene KEGG enrichment

Using the functional annotation tool of DAVID, the KEGG pathway enrichment analysis was carried out for the genes corresponding to the 1000 probes listed in the MaxRel. The enrichment results showed that these genes were significantly enriched in the energy metabolism pathways, including fatty acid metabolism, pentose and glucuronate interconversions, as well as starch and sucrose metabolism (**Table 3**). These results suggested that metabolism of nutrients may play critical role in the tumorigenesis of CRC.

**Table 3:** MaxRel table genes KEGG enrichment.

Term	KEGG ID	Count^a^	Percentage^b^	P-value	Benjamini Adjusted P-Value
Fatty acid metabolism	00071	11	1.2	8.4E-5	1.5E-2
Pentose and glucuronate interconversions	00040	7	0.8	3.0E-4	2.7E-2
Starch and sucrose metabolism	00500	10	1.1	6.6E-4	3.8E-2

a The number of genes belonging to a certain pathway.

b The percentage of genes belonging to a certain pathway account for all the genes underwent KEGG pathway analysis.

### Six candidate genes and shortest paths genes of KEGG enrichment

The KEGG pathway enrichment analysis was also performed on the 41 genes including the top six genes in the mRMR list and 35 genes in the shortest paths between these six genes with the functional annotation tool of DAVID. The enrichment result thus obtained showed that these genes were significantly enriched in the canonic cancer related pathways, such as prostate cancer, pathways in cancer, Wnt signaling pathway, cell cycle, colorectal cancer, thyroid cancer, and so on. It is instructive to note that among these pathways, some have been proved to be relevant to colorectal cancer including Wnt signaling pathway, cell cycle, colorectal cancer and insulin signaling pathway (**Table 4**).

**Table 4:** mRMR top six genes and shortest path genes KEGG enrichment.

Term	KEGG ID	Count^a^	Percentage^b^	P-value	Benjamini Adjusted P-Value
Prostate cancer	05215	8	19.5	3.80E-08	2.40E-06
Pathways in cancer	05200	10	24.4	2.60E-06	8.00E-05
Wnt signaling pathway	04310	6	14.6	3.00E-04	6.30E-03
Huntington's disease	05016	6	14.6	6.70E-04	1.10E-02
Notch signaling pathway	04330	4	9.8	8.80E-04	1.10E-02
Cell cycle	04110	5	12.2	1.50E-03	1.60E-02
Insulin signaling pathway	04910	5	12.2	2.00E-03	1.80E-02
Colorectal cancer	05210	4	9.8	4.70E-03	3.60E-02
Thyroid cancer	05216	3	7.3	6.20E-03	4.20E-02
Melanogenesis	04916	4	9.8	7.40E-03	4.60E-02

a The number of genes belonging to a certain pathway.

b The percentage of genes belonging to a certain pathway account for all the genes underwent KEGG pathway analysis.

### Overlap with cancer related gene list and two CRC related gene lists

We compiled 742 cancer-related genes from the following three different sources: Cancer Gene Census from the Sanger Centre, Atlas of Genetics and Cytogenetic in Oncology [21], and Human Protein Reference Database [22]. It was observed that 8 out of the 41 genes identified by us were proven to be cancer-related genes. Also, it was indicated by the Fisher's exact test that these 41 genes were significantly related to cancer (p-value = 0.0001908). See Supporting Information S5.

Moreover, we collected 438 genes that were differentially expressed between colorectal adenomas and normal mucosa from previous study [23]. Interestingly, the aforementioned 41 candidate genes identified by us had an overlap of 4 genes with the 438 genes, and the overlap was quite significant (p-value = 0.01057, Fisher's exact test). See Supporting Information S5.

Recently, the Boolean based systems biology approach was employed to identify 134 novel CRC related genes [24], of which three were identified by us in this study and the overlap was significant (p-value = 0.002017, Fisher's exact test). See Supporting Information S5.

## Discussion

### KEGG enrichment of MaxRel genes

The genes corresponding to the 1000 probes listed in the MaxRel table were significantly enriched in the energy metabolism pathways, including fatty acid metabolism, pentose and glucuronate interconversions, as well as starch and sucrose metabolism. It has been shown that diet has an important effect on the CRC development. Our finding is quite consistent with the fact that genetic polymorphisms influencing the metabolism of nutrients play an important role in the etiology of CRC and colorectal adenomatous polyps [62].

Multiple lines of evidences have indicated the implication or involvement of fat in the etiology of CRC [66]. The crucial role of fatty acids in numerous biological processes suggests that alteration in fatty acid metabolizing genes contributes to colon carcinogenesis [67]. It has been shown that starch and sucrose metabolism and pentose and glucuronateinterconversions were closely related to cancers. Christensen et al. [68] demonstrated that starch and sucrose metabolism and pentose and glucuronateinterconversions pathway were hypomethylated in isocitrate dehydrogenase mutant tumors. In addition, these two metabolic pathways were found to be significantly related to the risk of developing estrogen receptor-negative breast cancer [69].

A recent CRC disease-specific transcriptome research showed that starch and sucrose metabolism was one of the 7 common pathway significant differentially regulated using two different microarray platforms including Affymetrix HGU133 Plus2.0 array and the CRC disease specific array. Besides, fatty acid metabolism was identified as significantly differentially regulated pathway using colorectal disease specific array [70].

### Six candidate genes identified by mRMR, NNA and IFS

In this study, we have identified the following six genes: GUCA2B, PI16, CDH3, SPIB, BEST2, and HMGCLL1. Below, let us briefly discuss their relationships with colorectal cancer.

GUCA2B (uroguanylin) is an endogenous activator of the guanylate cyclase-2C receptor found to be down regulated 8-fold in adenoma, and its expression is detected in blood and urine [71].Therefore, GUCA2B could be regarded as a non-invasive biomarker for the early detection of CRC. In addition, the radio labeled uroguanylin analogs have been used for detection of CRC in vivo [72].

PI16 (Peptidase inhibitor 16) is detected within the testis, prostate, small intestine, colon, and ovary with immunohistochemical analyses [73]. Decrease of PI16 level was detected in prostate cancer [73] and gastric cancer [74]. Our result also showed that the expression of PI16 in colorectal adenocarcinoma was significant decreased compared with the adjacent non-tumor colorectal tissue, which was consistent with the result of the research in prostate cancer and gastric cancer. Since PI16 is not well characterized and so far there is no report whatsoever about PI16 in colorectal cancer etiology, our result implied that PI16 may become a promising biomarker for colorectal cancer early diagnosis.

CDH3 is a classical cadherin, the demethylation of which is frequently detected in the advanced CRC which was associated with the overexpression of CDH3 [75]. Besides CRC, CDH3 was also overexpressed in the majority of pancreatic cancer and gastric cancer, but not in their noncancerous counterparts or in normal tissues. Thus CDH3 was regarded as a novel tumor-associated antigen useful for immunotherapy and early diagnosis of gastric cancer and CRC [76].

SPIB is a transcription factor of the E-twenty-six (ETS) family, which is known to act as positive or negative regulators of gene expression. SPIB is an adenoma condition-specific down regulated gene and its expression underwent a striking decrease in CRC tissues indicating that SPIB may serve as potential markers of CRC invasiveness and metastasis [77].

BEST2 (also known as VMD2L1) encodes a protein of the bestrophin family. Both RT-PCR analyses and X-gal staining revealed tissue-restricted BEST2 and VMD2L2 abundantly expressed in colon [78], [79]. It has been show that BEST2 mediates bicarbonate transport by goblet cells in mouse colon [80]. Straub et al. [81] identified BEST2 as one of the methylation markers for early detection and prognosis of CRC. Therefore, BEST2 was expected to become a therapy target for CRC with demethylation agent.

HMGCLL1 has been show to be related to various cancers, such as pancreatic cancers [82], glioblastoma multiforme [83], breast and colorectal cancers [84]. HMGCLL1 is one of the genes containing somatic mutations in pancreatic cancer [82]. Though mutation in HMGCLL1 has been reported to be involved in these cancers, the specific mechanisms underlying remain to be elucidated.

### Shortest path genes

We totally identified 35 shortest paths genes. As we can see from **Table 2**, some shortest path genes such as TP53, EP300, CTNNB1 and GSK3B were not significant for CRC due to their universality in numerous cancers. However, these genes have been well documented to be relevant to CRC, and also their role in CRC has been well characterized [85]. Besides these genes, most of the other shortest genes listed in Table 2 were quite specific to CRC (p-value<0.05). Below, let us focus on the specific genes with the large betweenness values and discuss the relationship of such genes with CRC.

AR (androgen receptor) is a ligand dependent transcription factor, which is involved in the control of cellular proliferation and differentiation [86]. Several studies have provided supporting evidences for its involvement of sex steroid hormones (estrogens and androgens) in the etiology and progression of CRC [87]. AR protein has been shown to be expressed in normal colorectal mucosa and in most colorectal cancer [61], [62], supporting that CRC expressing the AR receptor may respond to mitogenic effects of the hormone. Moreover, somatic reductions of the androgen receptor CAG repeat occur frequently, through a pathway different from microsatellite instability and early during colon carcinogenesis. Apparent growth selection of cells harboring shortened AR alleles suggests that androgens contribute to colon carcinogenesis in a yet unknown way [61].

TBP (the TATA-binding protein) is a key eukaryotic transcription factor used by all three cellular RNA polymerases. Compared to normal colon epithelium, TBP expression is elevated in the case of human colon carcinomas. Both Ras-dependent and Ras-independent mechanisms mediate the increases of TBP expression in colon carcinoma cell lines. Thus, TBP may be a crucial component in dysregulated signaling for causing tumors [88].

MYOD1 promoter methylation occurs in various malignancies including CRC. MYOD1 promoter methylation was detectable in tumor and normal colorectal samples, but was significantly higher in tumor than in normal mucosa. Patients without MYOD1 hypermethylation showed significantly longer survival than those with hypermethylation. Therefore, MYOD1 hypermethylation plays an important role in CRC and may be a novel prognostic factor [89].

HMGCR (3-hydroxy-3-methylglutaryl coenzyme A reductase) is an enzyme that catalyzes the rate-limiting step of cholesterol biosynthesis. HMGCR alternative splicing of exon 13 is not only a biomarker, but also a determinant of statin efficacy, which is a class of cholesterol-lowering drugs that inhibit HMGCR. HMGCR was used not only for the treatment of hypercholesterolemia, but also as a chemopreventive agent for CRC [90]. A genetic test of HMGCR was utilized to determine in which patients cholesterol-lowering statin drugs might have the most benefit in reducing the risk of CRC. A recent research has found a genetic variant may affect the way of how statins control both colorectal cancer and cardiovascular disease risk [91].

KLK3 (also known as prostate-specific antigen, PSA) is a kallikrein-like serine protease that is a widely used biomarker for prostate cancer [92]. In addition to prostate cancer, breast, colon, ovarian, liver and kidney tumors can also produce KLK3 [93]. Recently, several other members of KLK family like KLK7 have shown promise as potential biomarkers for various cancers including colon cancer [94], [95], [96]. Thus, with the progress of research, KLK3 may become a biomarker for CRC as well.

CHD (Chromodomain helicase DNA-binding protein) is a regulator of the chromatin remodeling process. CHD4 expression was detected in gastric cancers and CRCs by immunohistochemistry. It has been reported that loss of CHD4 expression was observed in 56.4% of the gastric cancers and 55.7% of the CRCs. In addition, Frameshift mutation and loss of expression of CHD genes are common in gastric cancers and CRCs with MSI-H. These alterations might contribute to cancer pathogenesis by deregulating CHD-mediated chromatin remodeling [97].

MSMB encodesβ-microsemino protein, which is a proposed biomarker for prostate cancer [98]. Genome-wide association studies (GWAS) have identified a variant, rs10993994, on chromosome 10q11 which is associated with prostate cancer risk. So far, there is no report about MSMB in CRC etiology. However, the expression of MSMB was detected in colon epithelial cells by immunohistochemistry [99]. Thus, it may be a potential biomarker for colorectal cancer diagnosis although it is remained to be verified.

FOXJ3 is a member of Human Forkhead-box (FOX) gene family. It has been shown that genetic and epigenetic changes of FOX family genes as well as alterations occurring in target genes of FOX transcription factors family could lead to human disease including carcinogenesis [100]. Recently, Niittymaki et al. [101] identified a SNP, rs2761880, locates in the binding site of FOXJ3 in CRC. It has been proposed that many of the predisposition loci for CRC are involved in control of gene expression by targeting transcription factor binding sites. In addition, oligonucleotide microarray analysis of distinct gene expression patterns in CRC tissues harboring BRAF and K-ras mutations has shown that FOXJ3 was identified by PAM (Prediction analysis of microarrays) and the jackknife (or leave-one-out) cross validation as candidate to distinguish the mutant groups [102].

HDAC1 (Histone deacetylase 1) is involved in tumorigenesis through their regulation of cell proliferation, differentiation and survival. In cancer cells, HDAC1 represses the expression of tumor suppress genes such as p21/WAF1/CIP1 and Bax, leading to aberrant cell proliferation and cell viability [103]. HDAC1 and HDAC3 are overexpressed in colon cancer cells and in primary colon cancer, and siRNA (small interfering RNA) mediated silencing of HDAC1 and HDAC3 in colon cancer cells induced apoptosis [104].

MEF2C (myocyte enhancer factor 2C) is a member of the MEF2 family of transcription factors. Recently, MEF2C was identified as a potential oncogenic transcription factor associated with CRC [24]. Besides, it has been shown that MEF2C was hypermethylated. Also, it was indicated by the significantly down-regulated in colon cancer that MEF2C may play a role in CRC etiology [105].

NR1H3 is a transcription factor involved in lipid homeostasis and inflammation. Recent evidences indicated that miRNAs can bind to the 3′untranslatedregions (UTRs) of mRNAs and regulates their translation. Genetic polymorphisms can locate in miRNA binding sites. Thus, miRNA regulation may be influenced by polymorphisms on the 3′UTRs. NR1H3 was identified as a candidate gene that harboring polymorphic in miRNA target sites which was associated with risk of sporadic CRC [106]. The specific relationship between NR1H3 and CRC remains to be further elucidated.

### Overlap between selected genes and known cancer genes as well as known CRC related genes

Statistic test showed that the overlap between the 41 genes identified in our study and the 742 cancer-related genes we compiled was quite significant (p-value = 0.0001908). The KEGG analysis result of such 41 genes also implied that they were significantly enriched in cancer-related pathways (p-value = 8.00E-05). Taken together, it indicated that the 41 genes identified by us were closely associated with cancer. In addition, the overlaps of such 41 candidate genes with the previous (p-value = 0.01057) and recent (p-value = 0.002017) reported CRC biomarkers were significant. This suggested that the 41 candidate genes have the potential to be used as biomarkers for CRC diagnosis.

In addition, we compared the 41 genes identified by us with the top 41 genes in mRMR feature list and the top 41 differentially expressed genes identified by the traditional t-test method of R language [107]. See the Supporting Information S6 for such three sets of 41 genes. As can be seen from there, the 41 genes identified by us contain 8 cancer genes, which is more than 4 (p-value = 0.03965, proportion test) and 2 (p-value = 4.923e-05, proportion test) cancer genes than those contained in the 41 genes identified by mRMR and the 41 genes identified by the t-test, respectively (**Table 5**).

**Table 5:** The overlap between 41 genes identified from three different methods and 742 cancer genes.

	Overlap with 742 Cancer genes	p-value
Our 41 genes	8	
Top 41 mRMR genes	4	0.03965
Top 41 t-test genes	2	4.923e-05

### Functional similarity between selected genes and known CRC related genes

In this study, five gene sets were defined. The first gene set is our 41 selected genes. The second gene set is the top 41 mRMR genes. The third gene set is the top 41 t-test genes that have the smallest t-test p values. The second and third gene sets were from gene expression profiles alone. Our 41 gene were selected based on both gene expression profiles and protein interaction network. The fourth gene set is the 742 cancer genes mentioned above. The fifth gene set is the combined known CRC related genes of 742 cancer related genes, 438 genes from Sabates-Bellver's study [23]and 134 colorectal cancer related genes from Nagaraj's study [24]. These five gene sets can be found in the Supporting Information S6.

To compare the functional similarity between our selected genes and the known CRC related genes, we constructed their functional profiles using the −log10 of the hypergeometric test p value on Gene Ontology (GO) terms [1], [5], [108]. Then we calculated the Pearson correlation coefficient of their functional profiles [1], [109]. The functional similarities of the functional profiles for the five gene sets were shown in **Table 6**. Our 41 genes had greater functional similarity with the cancer genes and the known CRC genes than the genes identified from gene expression profiles alone: top 41 mRMR genes and top 41 t-test genes. This suggests that the genes selected by our method are more reliable than the genes identified from the gene expression profiles alone. Combining the gene expression profiles and protein interaction network together can improve the identification of disease genes.

**Table 6:** The functional similarity between our 41 genes and known colorectal cancer genes.

	Cancer genes	Colorectal cancer genes
Our 41 genes	0.606068*	0.491953*
Top 41 mRMR genes	0.163112*	0.244468*
Top 41 t-test genes	0.203573*	0.269548*

* Pearson correlation coefficient of functional profiles.

The reason why our method can generate more reliable results is because that the shortest pathway approach integrated here is based on all the information of genes from database, text mining, etc. that is quite stable and can avoid the false positives. In contrast to this, the method based on the gene expression data can cause lots of false positives. It is anticipated that our method may become a useful tool, or at least play a complementary role to the existing method, for identifying colorectal cancer genes.

It is instructive to point out that our method may have some limitations. This is because some hub genes that may simultaneously interact with lots of other genes can also occur in our shortest path and the randomly selected shortest paths, such as TP53 and EP300. Nevertheless, our method can provide a p-value to evaluate the significance that can be used to distinguish the hubs in the network background.

### Conclusion

We proposed a novel method to identify cancer related genes. We applied this method on CRC and identified 41 genes which had the most potential to be biomarker for CRC early diagnose. Statistic test and KEGG analysis showed that the 41 candidate genes identified in our study are not only closely related to cancer but also have great potential to become biomarker for CRC diagnosis. In addition, the 41 candidate genes contain more cancer genes than the genes identified from gene expression profiles alone, and functional similarity analysis revealed that our genes had greater functional similarity with the reported CRC genes than the genes identified from gene expression profiles alone. We believe that our method may be helpful (or at least play a stimulative role) for predicting novel cancer related genes, and that it might have the potential applicability for the cancer research.

## Supporting Information

Supporting Information S1 **The cancer-related gene list and the two colorectal cancer-related gene lists.**
(XLS)

Supporting Information S2 **The MaxRel features table and mRMR features table.**
(XLS)

Supporting Information S3 **Feature numbers and the first order accuracy which the IFS curve plot was based on.**
(XLS)

Supporting Information S4 **The 15 shortest paths with the lowest cost presented with protein and gene, respectively.**
(DOC)

Supporting Information S5 **The overlap between the 41 candidate genes and the three other datasets and the corresponding Fisher's exact test.**
(DOC)

Supporting Information S6 **Five gene sets.** First gene set is our 41 selected genes. The second gene set is the top 41 mRMR genes. The third gene set is the top 41 t-test genes that have the smallest t-test p values. The second and third gene sets were from gene expression profiles alone. Our 41 gene were selected based on both gene expression profiles and protein interaction network. The fourth gene set is the 742 cancer genes. The fifth gene set is the combined known colorectal cancer related genes.
(XLS)
