# Supplementary material for: Identification of Colorectal Cancer Related Genes with mRMR and Shortest Path in Protein-Protein Interaction Network
Source: PLoS One. 2012 Apr 4;7(4):e33393. doi: 10.1371/journal.pone.0033393 (PMC3319543; doi:10.1371/journal.pone.0033393)
Supplement: Supporting Information S4 — The 15 shortest paths with the lowest cost presented with protein and gene, respectively. (DOC) [file pone.0033393.s004.doc]

Shortest path and lowest cost presented with protein

ENSP00000264012->ENSP00000344456->ENSP00000363822->ENSP00000314151->ENSP00000351363->ENSP00000362778 362

ENSP00000264012->ENSP00000344456->ENSP00000363822->ENSP00000361066->ENSP00000263253->ENSP00000250003->ENSP00000396219->ENSP00000354620->ENSP00000361662 255

ENSP00000264012->ENSP00000344456->ENSP00000363822->ENSP00000399968->ENSP00000419692->ENSP00000342470->ENSP00000363868->ENSP00000354476->ENSP00000344741->ENSP00000287936->ENSP00000381654 190

ENSP00000264012->ENSP00000344456->ENSP00000324806->ENSP00000269305->ENSP00000362649->ENSP00000349508->ENSP00000417884->ENSP00000042931 324

ENSP00000264012->ENSP00000344456->ENSP00000324806->ENSP00000269305->ENSP00000230354->ENSP00000270632 148

ENSP00000362778->ENSP00000351363->ENSP00000314151->ENSP00000363822->ENSP00000361066->ENSP00000263253->ENSP00000250003->ENSP00000396219->ENSP00000354620->ENSP00000361662 491

ENSP00000362778->ENSP00000351363->ENSP00000314151->ENSP00000363822->ENSP00000399968->ENSP00000419692->ENSP00000342470->ENSP00000363868->ENSP00000354476->ENSP00000344741->ENSP00000287936->ENSP00000381654 426

ENSP00000362778->ENSP00000351363->ENSP00000314151->ENSP00000363822->ENSP00000268712->ENSP00000362649->ENSP00000349508->ENSP00000417884->ENSP00000042931 561

ENSP00000362778->ENSP00000351363->ENSP00000314151->ENSP00000363822->ENSP00000361066->ENSP00000262367->ENSP00000269305->ENSP00000230354->ENSP00000270632 386

ENSP00000361662->ENSP00000354620->ENSP00000396219->ENSP00000250003->ENSP00000263253->ENSP00000206249->ENSP00000254227->ENSP00000342470->ENSP00000363868->ENSP00000354476->ENSP00000344741->ENSP00000287936->ENSP00000381654 318

ENSP00000361662->ENSP00000354620->ENSP00000396219->ENSP00000250003->ENSP00000263253->ENSP00000329357->ENSP00000362649->ENSP00000349508->ENSP00000417884->ENSP00000042931 451

ENSP00000361662->ENSP00000354620->ENSP00000396219->ENSP00000250003->ENSP00000263253->ENSP00000269305->ENSP00000230354->ENSP00000270632 275

ENSP00000381654->ENSP00000287936->ENSP00000344741->ENSP00000354476->ENSP00000363868->ENSP00000342470->ENSP00000419692->ENSP00000348827->ENSP00000348551->ENSP00000362649->ENSP00000349508->ENSP00000417884->ENSP00000042931 388

ENSP00000381654->ENSP00000287936->ENSP00000344741->ENSP00000265565->ENSP00000297146->ENSP00000348069->ENSP00000348986->ENSP00000304895->ENSP00000353483->ENSP00000269305->ENSP00000230354->ENSP00000270632 212

ENSP00000042931->ENSP00000417884->ENSP00000349508->ENSP00000362649->ENSP00000269305->ENSP00000230354->ENSP00000270632 344

Shortest path and lowest cost presented with genes

CDH3->CTNNB1->AR->KLK3->MSMB->PI16 362

CDH3->CTNNB1->AR->NCOA3->EP300->MYOD1->MEF2C->FOXJ3->GUCA2B 255

CDH3->CTNNB1->AR->NCOA2->RXRA->NR1H3->ABCA1->SREBF2->INSIG1->HMGCR->HMGCLL1 190

CDH3->CTNNB1->GSK3B->TP53->HDAC1->CHD4->TRIM27->BEST2 324

CDH3->CTNNB1->GSK3B->TP53->TBP->SPIB 148

PI16->MSMB->KLK3->AR->NCOA3->EP300->MYOD1->MEF2C->FOXJ3->GUCA2B 491

PI16->MSMB->KLK3->AR->NCOA2->RXRA->NR1H3->ABCA1->SREBF2->INSIG1->HMGCR->HMGCLL1 426

PI16->MSMB->KLK3->AR->NCOR1->HDAC1->CHD4->TRIM27->BEST2 561

PI16->MSMB->KLK3->AR->NCOA3->CREBBP->TP53->TBP->SPIB 386

GUCA2B->FOXJ3->MEF2C->MYOD1->EP300->ESR1->NR0B2->NR1H3->ABCA1->SREBF2->INSIG1->HMGCR->HMGCLL1 318

GUCA2B->FOXJ3->MEF2C->MYOD1->EP300->SP1->HDAC1->CHD4->TRIM27->BEST2 451

GUCA2B->FOXJ3->MEF2C->MYOD1->EP300->TP53->TBP->SPIB 275

HMGCLL1->HMGCR->INSIG1->SREBF2->ABCA1->NR1H3->RXRA->THRB->NCOR2->HDAC1->CHD4->TRIM27->BEST2 388

HMGCLL1->HMGCR->INSIG1->SCAP->GPR85->SREBF1->INS-IGF2->IRS1->MAPK8->TP53->TBP->SPIB 212

BEST2->TRIM27->CHD4->HDAC1->TP53->TBP->SPIB 344
