# Supplementary material for: Identification of Colorectal Cancer Related Genes with mRMR and Shortest Path in Protein-Protein Interaction Network
Source: PLoS One. 2012 Apr 4;7(4):e33393. doi: 10.1371/journal.pone.0033393 (PMC3319543; doi:10.1371/journal.pone.0033393)
Supplement: Supporting Information S5 — The overlap between the 41 candidate genes and the three other datasets and the corresponding Fisher's exact test. (DOC) [file pone.0033393.s005.doc]

Overlap with cancer related gene list and two colorectal cancer related gene lists

| symbol | list 1 | list 2 | list 3 |
| --- | --- | --- | --- |
| AR | √ |  |  |
| TP53 | √ |  |  |
| TBP |  |  |  |
| MYOD1 |  |  |  |
| EP300 | √ |  |  |
| HMGCR |  |  |  |
| KLK3 |  |  |  |
| CTNNB1 | √ |  |  |
| INSIG1 |  |  |  |
| CHD4 |  |  |  |
| MSMB |  |  |  |
| FOXJ3 |  |  |  |
| HDAC1 |  |  |  |
| MEF2C |  |  | √ |
| NR1H3 |  |  |  |
| SREBF2 |  |  |  |
| ABCA1 | √ |  |  |
| NCOA3 |  |  |  |
| RXRA |  |  |  |
| GSK3B |  |  |  |
| NCOA2 | √ |  |  |
| ESR1 |  |  |  |
| NR0B2 |  |  |  |
| CREBBP | √ |  |  |
| SCAP |  |  |  |
| NCOR1 |  |  |  |
| GPR85 |  |  |  |
| IRS1 |  |  |  |
| SP1 |  |  |  |
| SREBF1 |  |  |  |
| NCOR2 |  |  |  |
| THRB |  | √ |  |
| INS-IGF2 |  |  |  |
| MAPK8 |  |  |  |
| CDH3 |  | √ |  |
| PI16 |  |  |  |
| GUCA2B |  | √ | √ |
| HMGCLL1 |  |  |  |
| BEST2 |  |  |  |
| SPIB |  | √ | √ |
| TRIM27 | √ |  |  |
|  |  |  |  |

List 1: Cancer related gene list

List 2: colorectal cancer related gene list from Sabates-Bellver et al.

List 3: colorectal cancer related gene list from Nagaraj et al.

“√” represents overlap between the two gene sets

Overlap with list 1

|  |  | cancer related | |  |
| --- | --- | --- | --- | --- |
|  |  | yes | no | total |
| identified by us | yes | 8 | 33 | 41 |
|  | no | 734 | 17716 | 18450 |
| total |  | 742 | 17749 | 18491 |

Fisher’s exact test (p-value= 0.0001908)

742 cancer related genes were retrieved from Cancer Gene Census of the Sanger Centre, Atlas of Genetics and Cytogenetic in Oncology and Human Protein Reference Database

18491 is the total gene number in Illumina Ref-8 whole-genome expression BeadChip

Overlap with list 2

|  |  | differentially expressed genes | |  |
| --- | --- | --- | --- | --- |
|  |  | yes | no | total |
| identified by us | yes | 4 | 37 | 41 |
|  | no | 434 | 20352 | 20786 |
| total |  | 438 | 20389 | 20827 |

Fisher’s exact test (p-value=0.01057)

20827 is the total gene number analyzed in the study of Sabates-Bellver et al.

Overlap with list 3

|  |  | novel CRC related genes | |  |
| --- | --- | --- | --- | --- |
|  |  | yes | no | total |
| identified by us | yes | 3 | 38 | 41 |
|  | no | 131 | 21720 | 21851 |
| total |  | 134 | 21758 | 21892 |

Fisher’s exact test (p-value=0.002017)

21892 is the total gene number analyzed in the study of Nagaraj et al.

1. Huret JL, Dessen P, Bernheim A (2003) Atlas of Genetics and Cytogenetics in Oncology and Haematology, year 2003. Nucleic Acids Res 31: 272-274.

2. Keshava Prasad TS, Goel R, Kandasamy K, Keerthikumar S, Kumar S, et al. (2009) Human Protein Reference Database--2009 update. Nucleic Acids Res 37: D767-772.

3. Sabates-Bellver J, Van der Flier LG, de Palo M, Cattaneo E, Maake C, et al. (2007) Transcriptome profile of human colorectal adenomas. Mol Cancer Res 5: 1263-1275.

4. Nagaraj SH, Reverter A (2011) A Boolean-based systems biology approach to predict novel genes associated with cancer: Application to colorectal cancer. BMC Syst Biol 5: 35.

5. Hinoue T, Weisenberger DJ, Lange CP, Shen H, Byun HM, et al. (2011) Genome-scale analysis of aberrant DNA methylation in colorectal cancer. Genome Res.
